# DUET: a server for predicting effects of mutations on protein stability using an integrated computational approach

**DOI:** 10.1093/nar/gku411

**Published:** 2014-05-14

**Authors:** Douglas E.V. Pires, David B. Ascher, Tom L. Blundell

**Affiliations:** 1 Department of Biochemistry, University of Cambridge, Cambridge, CB2 1GA, UK; 2 ACRF Rational Drug Discovery Centre and Biota Structural Biology Laboratory, St Vincents Institute of Medical Research, Fitzroy, VIC 3065, Australia

## Abstract

Cancer genome and other sequencing initiatives are generating extensive data on non-synonymous single nucleotide polymorphisms (nsSNPs) in human and other genomes. In order to understand the impacts of nsSNPs on the structure and function of the proteome, as well as to guide protein engineering, accurate *in silico*methodologies are required to study and predict their effects on protein stability. Despite the diversity of available computational methods in the literature, none has proven accurate and dependable on its own under all scenarios where mutation analysis is required. Here we present DUET, a web server for an integrated computational approach to study missense mutations in proteins. DUET consolidates two complementary approaches (mCSM and SDM) in a consensus prediction, obtained by combining the results of the separate methods in an optimized predictor using Support Vector Machines (SVM). We demonstrate that the proposed method improves overall accuracy of the predictions in comparison with either method individually and performs as well as or better than similar methods. The DUET web server is freely and openly available at http://structure.bioc.cam.ac.uk/duet.

## INTRODUCTION

In this era of high-throughput data generation, the ability to predict accurately the impacts of non-synonymous single nucleotide polymorphisms (nsSNPs) on protein stability is an essential tool for understanding the effects of human genome variation (1), particularly with respect to personalized medicine and the mechanisms of variable drug response in humans (2). The enormous amount of data being generated from cancer genome and other sequencing initiatives (3,4) requires an accurate and scalable computational approach to understanding structural effects of mutations and correlating them with disease on the scale of the whole proteome (5). Such a computational approach should also be useful in the development of engineered proteins with improved, modified or optimized functions (6).

Over the past fifteen years, several different *in silico* methods for predicting the influence of mutations on protein stability have been proposed based on various evolutionary and physical chemical hypotheses (7–15), but none has proven on its own to be accurate in all situations where mutational analysis is required. For this reason, one may expect to obtain a more accurate prediction by combining methods that are based on different paradigms and that exploit different protein structural properties (16), in order to reach a consensus on the understanding of mutation effects by an integrated computational approach. As highlighted in (15), the methods mCSM and SDM (7,14) are complementary since they measure different properties and are built upon different perspectives; a combined predictor should therefore improve overall performance.

Here, we present DUET, an integrated computational approach for predicting effects of missense mutations on protein stability. DUET combines mCSM and SDM in a consensus prediction, by consolidating the results of the separate methods in an optimized predictor using Support Vector Machines (SVMs) trained with Sequential Minimal Optimization (17).

DUET was trained on a low-redundancy data set of mutations with available experimental thermodynamic data derived from the ProTherm database (18) and validated with blind test sets, achieving a Pearsons correlation coefficient of up to 0.74 during training and 0.71 in the test set (0.82 and 0.79 after 10% outlier removal, respectively). We demonstrate that DUET improves overall accuracy of the predictions in comparison with either method on its own. We also show that DUET, by selectively combining two methods, significantly outperforms another integrated approach that combines seven methods (16). A web server for DUET is available at http://bleoberis.bioc.cam.ac.uk/duet.

## MATERIALS AND METHODS

### SDM

The method SDM, introduced in (7,14), relies on amino acid propensities derived from environment-specific substitution tables for homologous protein families that feed a statistical potential energy function and encompass an evolutionary view of the constraints from the immediate residue environment. The approach compares amino acid propensities for the wild-type and mutant proteins in the folded and unfolded states in order to estimate the free energy differences between wild type and mutant. The website is at: http://www-cryst.bioc.cam.ac.uk/sdm/sdm.php.

### mCSM

mCSM is a machine learning method to predict the effects of missense mutations based on structural signatures (15). The mCSM signatures were derived from the graph-based concept of Cutoff Scanning Matrix (CSM) (19), originally proposed to represent network topology by distance patterns in the study of biological systems. mCSM uses a graph representation of the wild-type residue environment to extract geometric and physicochemical patterns (the last represented in terms of pharmacophores) that are then used to represent the 3D chemical environment during supervised learning. These signatures have been successfully applied in a range of tasks including protein structural classification and function prediction (20), as well as large-scale receptor-based protein ligand prediction (21). The mCSM website is available at: http://structure.bioc.cam.ac.uk/mcsm.

### DUET-Integrated Computational Approach

**Figure 1.**
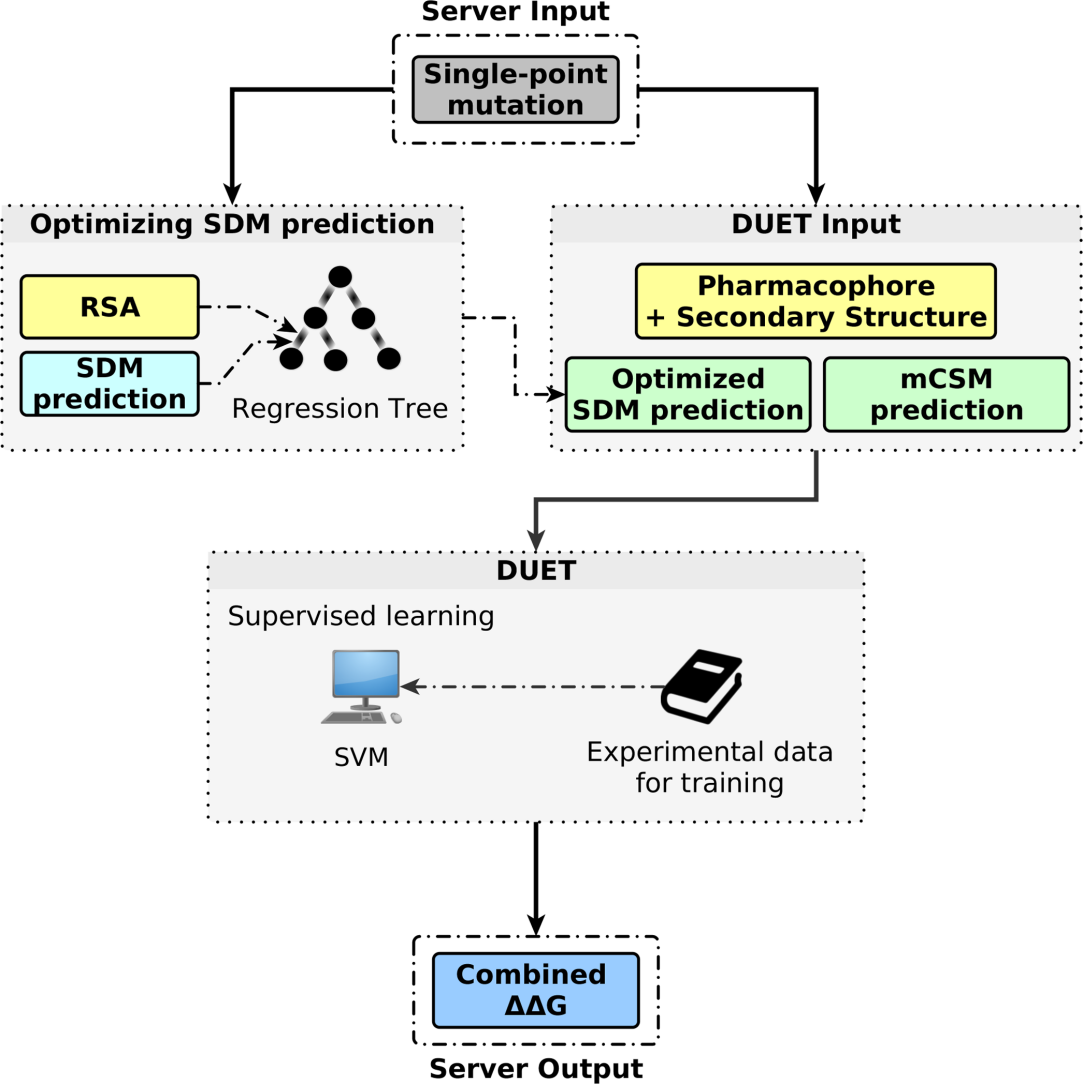
DUET workflow for obtaining a consensus prediction for a single point mutation. The grey and the blue boxes denote the server's input and output, respectively. Green boxes denote intermediate prediction values used by DUET and yellow boxes denote complementary information used to optimize SDM prediction or by DUET.

Figure 1 shows the workflow of the developed methodology. Given a single point mutation in a protein structure, DUET calculates a combined/consensus prediction by combining the predictions from two methods (mCSM and SDM) in a non-linear way, using SVM regression with a Radial Basis Function kernel (22).

In order to do so, complementary information regarding the mutation, such as secondary structure (used by SDM) and a pharmacophore vector that accounts for the changes between wild-type and mutant residue (used by mCSM) are also calculated and used by DUET. As described previously (15), the pharmacophore vector is obtained by comparing the frequency of eight possible atom characteristics between wild-type and mutant residues (hydrophobic, positive, negative, hydrogen acceptor, hydrogen donor, aromatic, sulphur and neutral).

As a filtering step, residue relative solvent accessibility (RSA) is used to optimize the standard SDM predictions using a regression model tree before combining it with mCSM. The M5P algorithm (23) was used to generate the regression tree which improved the SDM performance on the blind test from *r* = 0.56 to *r* = 0.62.

Finally, the mCSM and optimized SDM predictions, together with secondary structure from SDM and the pharmacophore vector from mCSM are fed to the SVM algorithm, generating a combined output from a supervised learning scheme. The experimental thermodynamic data for each mutation in training and test sets are used to evaluate the accuracy of the combined method.

## WEB SERVER

### Input

In order to run a prediction on the DUET server, the user submits a PDB structure or 4-letter code of the wild-type protein of interest, as well as the mutation information (residue position, wild-type and mutant residues codes in one-letter format) and chain identifier. Users also have the option to perform systematic mutations of a particular residue to all 19 possible mutants. DUET supports nuclear magnetic resonance structures but only the first model will be taken into account. Users are encouraged to submit PDB files with a single chain with the exception of cases of proteins that fold upon binding (coupled folding and binding of intrinsically disordered proteins (24)). A help page to assist users on how to run and interpret the results of the predictions is available on the top navigation bar.

### Output

**Figure 2.**
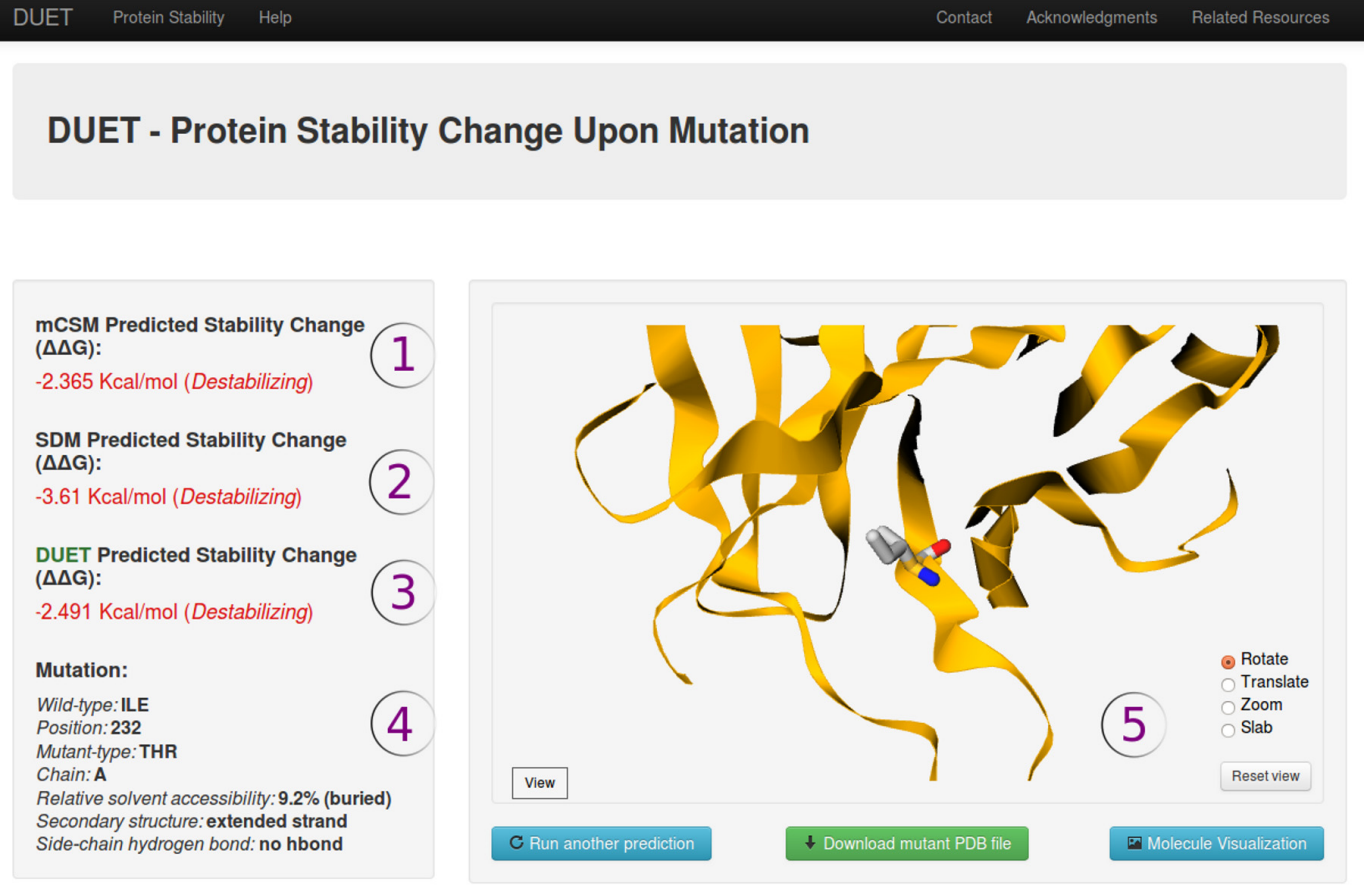
Result page for DUET prediction. The results display the predicted change in folding free energy upon mutation (ΔΔ*G* in kcal/mol). A positive value (and red writing) corresponds to a mutation predicted as destabilizing; while a negative sign (and blue writing) corresponds to a mutation predicted as stabilizing. The information displayed include the mCSM (i) and SDM (ii) individually predicted protein stability changes, the combined DUET prediction (iii), a structural summary of the mutation highlighting the wild-type residue and position number, the mutation and its 3D environment (iv). The protein and mutation can also be visualized (v), or a PDB file of the mutant downloaded for viewing in your preferred molecular visualization software.

As shown in Figure 2, the server displays in the output page the predictions from the individual methods, the combined/consensus prediction obtained by DUET and an interactive visualization of the uploaded PDB file via GLMol. This interface allows the user to visualize the protein with molecules represented in several ways, such as ‘cartoon’, ‘ball and stick’ and ‘spheres’ as well as to take snapshots. The predicted results are expressed as the variation in Gibbs Free Energy (ΔΔ*G*) and negative values denote destabilizing mutations. Complementary information such as residue relative solvent accessibility (RSA, calculated using the Richards method (25)), side-chain hydrogen bond satisfaction and secondary structure (programme SSTRUC) are calculated and shown. The user also has the option of downloading the structure of the mutant protein generated by the programme ANDANTE (26), as required for the method SDM.

## VALIDATION

### Mutation Data sets

DUET's regression model was trained on data for mutations derived from the ProTherm database (18) and used in a previous study (15). The training set is formed by 2297 randomly selected mutations drawn from the S2848 data set used by the PoPMuSiC method (13). To minimize the risk of overfitting, two blind test sets were devised to validate the method. The first data set was composed of 351 non-redundant mutations at position level, meaning that mutations in a given position are either in the training or test set exclusively. More information about the data sets used can be found in Section 1 in Supplementary Material. In order to perform a comparative test between DUET and iStable (16), we used a dataset of mutations on the p53 protein, a transcription factor whose loss of function is correlated with tumourigenesis which was assembled in a previous study (15). This data set contained 42 mutations within the DNA binding domain of the tumour suppressor p53 protein with experimentally characterized thermodynamic effects available in the scientific literature. None of these mutations was present in the training set.

## RESULTS

**Figure 3.**
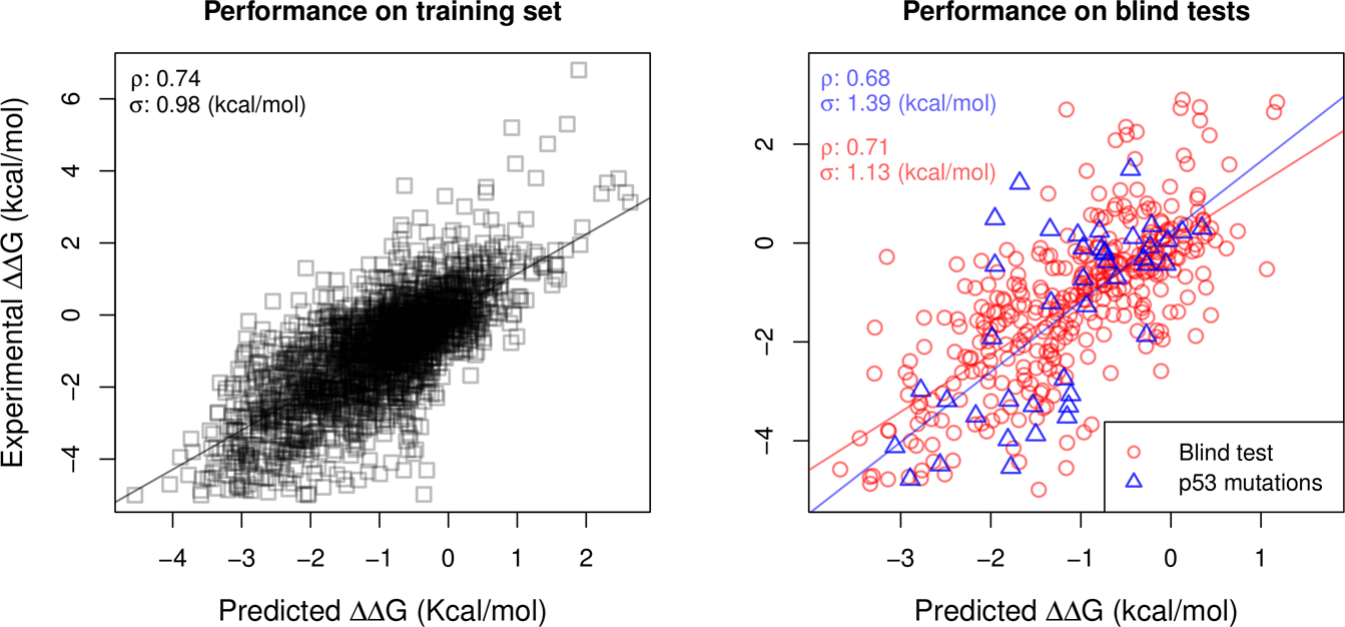
Regression analysis between experimental and predicted stability changes by DUET. The left graph show the performance of DUET during training while the right graph shows the predictive performance in two different blind test sets. Pearson's correlation coefficient (*r*) and standard error (*σ*) are also shown for each data set.

Figure 3 shows regression analysis for the stability predictions generated by DUET in comparison with the experimentally measured variation in stability for the considered data sets. During training, DUET achieved a Pearson's correlation coefficient of *r* = 0.74 with a standard error of *σ* = 0.98 kcal/mol, significantly better than mCSM (*r* = 0.69, *σ* = 1.06 kcal/mol. See Section 2 in Supplementary Material). Furthermore, a correlation of *r* = 0.82 with standard error of *σ* = 0.72 kcal/mol is obtained after 10% outlier removal. In the first blind test set of 351 non-redundant mutations, DUET achieved a correlation of *r* = 0.71 (*σ* = 1.13 kcal/mol, which is considerably higher than the performance of either method individually (*r* = 0.56 and *r* = 0.67 for SDM and mCSM, respectively. See Section 2 in Supplementary Material). The correlation in 90% of the data set peaks at *r* = 0.79 (*σ* = 0.84 kcal/mol).

In order to compare DUET with iStable (16), a recently proposed integrated computational approach, a blind test with p53 mutations was devised. iStable is a meta-predictor that combines seven different methods using SVM algorithm, and integrates complementary information such as residue solvent accessibility, secondary structure and sequence information.

Table 1 shows the comparative results between the computationally integrated approaches DUET and iStable, as well as mCSM and SDM. Even though iStable relies on the predictions of seven different methods, the approach achieved a correlation coefficient of only *r* = 0.49, which is inconsistent with the correlation of *r* = 0.86 that the authors report during cross-validation. In contrast, DUET achieves a *r* = 0.68 (*σ* = 1.40 kcal/mol), which is consistent with the methods performance during training and blind test validation. By removing 10% of outliers (only three mutations), DUET's correlation coefficient rises to *r* = 0.77 and standard error drops to *σ* = 1.12 kcal/mol, in comparison with a correlation of *r* = 0.64 (*σ* = 1.37 kcal/mol) achieved by iStable.

**Table 1.** Comparative prediction performance of methods on P53 data set

Method	Pearson's coefficient^a^	Standard error kcal/mol^a^
mCSM	0.68 / 0.72	1.40 / 1.20
SDM	0.52 / 0.64	1.61 / 1.32
iStable	0.49 / 0.64	1.59 / 1.37
**DUET**	**0.68 / 0.76**	**1.39 / 1.13**

^a^ The two values given per column correspond respectively to the whole validation set of 42 mutants and the results after removing 10% of the outliers.

## CONCLUSIONS

DUET is an accurate, free and easy-to-use bioinformatics web server created for experts and non-experts alike who are interested in gaining insight into the effects of nsSNPs on protein stability. It integrates two complementary methods into a consensus/optimized prediction, as a way to leverage the best of SDM, a statistical potential energy function that relies on substitution tables derived from homologous protein families which incorporates constraints on residue environments during evolution, and mCSM, a machine learning algorithm that takes into account the residue 3D phsycochemical environment summarized as a graph-based structural signature. DUET is a valuable tool for a wide variety of applications, ranging from protein stability modulation to understanding the role of mutations in diseases.

## SUPPLEMENTARY DATA

Supplementary Data are available at NAR Online including [1–6].

Supplementary Data
