# Supplementary material for: DUET: a server for predicting effects of mutations on protein stability using an integrated computational approach
Source: Nucleic Acids Res. 2014 May 14;42(Web Server issue):W314–9. doi: 10.1093/nar/gku411 (PMC4086143; doi:10.1093/nar/gku411)
Supplement: Supplementary Data [file supp_42_W1_W314__index.html]

Supplementary Data 

# DUET: a server for predicting effects of mutations on protein stability using an integrated computational approach

## Supplementary Data

**Files in this Data Supplement:**

- SUPPLEMENTARY DATA
