# Supplementary material for: DUET: a server for predicting effects of mutations on protein stability using an integrated computational approach
Source: Nucleic Acids Res. 2014 May 14;42(Web Server issue):W314–9. doi: 10.1093/nar/gku411 (PMC4086143; doi:10.1093/nar/gku411)
Supplement: Supplementary Data [file supp_gku411_nar-00587-web-b-2014-File010.pdf]

DUET: a server for predicting effects of mutations on protein stability using an integrated computational approach

Douglas E. V. Pires*, David B. Ascher, Tom L. Blundell*

Supplementary Material

1 Data sets

Training and test sets: DUET was trained and tested using data sets derived from the S2648 data set used by the PoPMuSiC method [1] and in a previous study [2], which represents a reduced set of single-point mutations with available experimental thermodynamic data in the ProTherm database [3]. The original data set comprises 2648 mutations in 131 different globular proteins. The effect of the mutations on protein stability is denoted by the difference in Gibbs free energy (ΔΔG), given in Kcal/mol. Experimental conditions, such as temperature and pH are also given. The distribution of these conditions for the S2648 data set is shown in Figure 1.

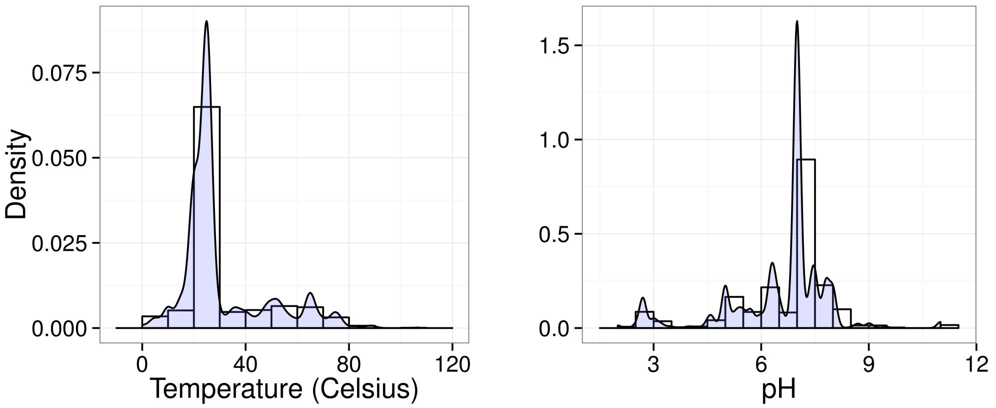

Figure 1: Histogram and density plots for the experimental conditions (temperature and pH) of the mutation.

In order to minimize redundancy and avoid biases during learning, low-redundancy training and test sets (in position level) were derived from the S2648 data set.
The training set is composed by 2297 randomly selected mutations from the S2648 data set, comprising 126 protein structures. To minimize the risk of overfitting, two blind test sets were devised to validate the method. The first data set is composed by the remaining 351 mutations (which accounts for 60 different protein structures). This division was done at position level, meaning that mutations in a given position of a protein are either in the training or test set exclusively . The distribution of mutations per protein is shown in Figure 2. These data sets were first proposed and used in a previous study [2].
The second data set, also proposed previously [2], was used to perform a comparative test between DUET and iStable [4]. It is composed by mutations of the p53 protein, a transcription factor whose loss of function is correlated with tumorigenesis. This data set contained 42 mutations within the DNA binding domain of the tumour suppressor p53 protein with experimentally characterized thermodynamic effects available in the scientific literature. None of these mutations was present in the training set.
The data sets described here are freely available at http://structure.bioc.cam.ac.uk/mcsm/data.

*Email: dpires@dcc.ufmg.br; Correspondence may also be addressed to tlb20@cam.ac.uk

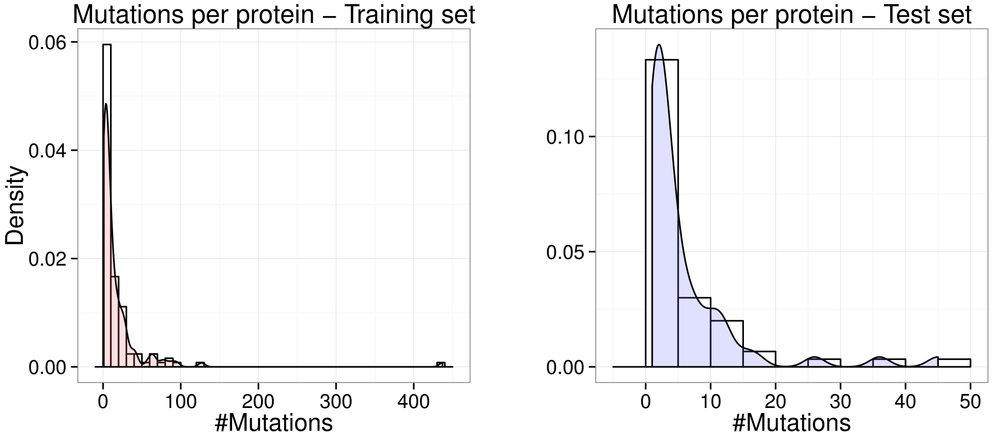

Figure 2: Histogram and density plots for the number of single-point mutations per protein in training and test sets.

2 Analysis of Performance Enhancement Achieved by Integration

Using Hotelling's t-test [5] and Steigers Z-test [6] for correlated correlations within a sample, DUET performed significantly better than SDM (two-tailed $p < 0.001$) on the blind test. DUET also outperformed mCSM in the training set (two-tailed $p < 0.0005$), but unfortunately the test set was too small to see significance.

References

[1] Y. Dehouck, A. Grosfils, B. Folch, D. Gilis, P. Bogaerts, and M. Rooman. Fast and accurate predictions of protein stability changes upon mutations using statistical potentials and neural networks: PoPMuSiC-2.0. *Bioinformatics*, 25(19):2537–2543, 2009.

[2] D. E. V. Pires, D. B. Ascher, and T. L. Blundell. mCSM: predicting the effects of mutations in proteins using graph-based signatures. *Bioinformatics*, 30(3):335–342, 2014.

[3] M. D. S. Kumar, K. A. Bava, M. M. Gromiha, P. Prabakaran, K. Kitajima, H. Uedaira, and A. Sarai. ProTherm and ProNIT: thermodynamic databases for proteins and protein–nucleic acid interactions. *Nucleic Acids Res.*, 34(Suppl 1):D204–D206, 2006.

[4] C. Chen, J. Lin, and Y. Chu. iStable: off-the-shelf predictor integration for predicting protein stability changes. *BMC Bioinf.*, 14(Suppl 2):S5, 2013.

[5] H. Hotelling. *The generalization of Students ratio*. Springer, 1992.

[6] J. H. Steiger. Tests for comparing elements of a correlation matrix. *Psychological Bulletin*, 87(2):245, 1980.
